# Time trends in mental health indicators during the initial 16 months of the COVID-19 pandemic in Denmark

**DOI:** 10.1186/s12888-021-03655-8

**Published:** 2022-01-10

**Authors:** Michelle T. Pedersen, Thea O. Andersen, Amy Clotworthy, Andreas K. Jensen, Katrine Strandberg-Larsen, Naja H. Rod, Tibor V. Varga

**Affiliations:** 1 Section of Epidemiology, Department of Public Health, University of Copenhagen, Copenhagen, Denmark; 2 Section of Biostatistics, Department of Public Health, University of Copenhagen, Copenhagen, Denmark; 3 Bartholinsgade 6Q, DK-1356 Copenhagen, Denmark

**Keywords:** COVID-19, Mental health, Mental illness, Anxiety, Loneliness, Worries, Quality of life, Prospective, Longitudinal, Time-series, Trend

## Abstract

**Background:**

The COVID-19 pandemic and its associated national lockdowns have been linked to deteriorations in mental health worldwide. A number of studies analysed changes in mental health indicators during the pandemic; however, these studies generally had a small number of timepoints, and focused on the initial months of the pandemic. Furthermore, most studies followed-up the same individuals, resulting in significant loss to follow-up and biased estimates of mental health and its change. Here we report on time trends in key mental health indicators amongst Danish adults over the course of the pandemic (March 2020 - July 2021) focusing on subgroups defined by gender, age, and self-reported previously diagnosed chronic and/or mental illness.

**Methods:**

We used time-series data collected by Epinion (N=8,261) with 43 timepoints between 20 March 2020 and 22 July 2021. Using a repeated cross-sectional study design, independent sets of individuals were asked to respond to the Copenhagen Corona-Related Mental Health questionnaire at each timepoint, and data was weighted to population proportions. The six mental health indicators examined were loneliness, anxiety, social isolation, quality of life, COVID-19-related worries, and the mental health scale. Gender, age, and the presence of previously diagnosed mental and/or chronic illness were used to stratify the population into subgroups for comparisons.

**Results:**

Poorer mental health were observed during the strictest phases of the lockdowns, whereas better outcomes occurred during reopening phases. Women, young individuals (<34 yrs), and those with a mental- and/or chronic illness demonstrated poorer mean time-series than others. Those with a pre-existing mental illness further had a less reactive mental health time-series. The greatest differences between women/men and younger/older age groups were observed during the second lockdown.

**Conclusions:**

People with mental illness have reported disadvantageous but stable levels of mental health indicators during the pandemic thus far, and they seem to be less affected by the factors that result in fluctuating time-series in other subgroups.

**Supplementary Information:**

The online version contains supplementary material available at 10.1186/s12888-021-03655-8.

## Introduction

On 11 March 2020, the World Health Organization (WHO) declared the novel Coronavirus-Disease-2019 (COVID-19) a global pandemic [1]. Given the lack of treatment and vaccines available at the time, governments in most countries adopted specific mitigation strategies to limit physical contact between individuals [2, 3]. The primary aim of these implemented restrictions was to contain the spread of the virus, thereby preventing healthcare systems to break down [3, 4]. As a consequence, pandemic-related restrictions, including lockdowns and physical distancing, have separated individuals from their regular social networks and limited their freedom of movement to varying degrees. Furthermore, the communication of information about the restrictive guidelines seems to have introduced uncertainties about the future [5–8]. While isolation is recommended to limit the spread of the virus, humans, who are inherently social, are not used to prolonged states of isolation [9]. Early evidence indicated acute deteriorations in the mental health of populations in many countries following the initial lockdowns [10, 11]. Conversely, as countries first began to reopen and/or restrictions were relaxed around May – July 2020, studies found that various mental health indicators simultaneously improved [5, 12, 13]. To date, however, no high-resolution longitudinal or time-series studies have tracked multiple mental health indicators over the entire course of the COVID-19 pandemic.

A growing body of literature is investigating the mental health consequences of the COVID-19 pandemic amongst different population subgroups. Of note, women have been shown to report higher levels of loneliness, depression, anxiety, and stress during the pandemic than men [14]. Furthermore, most studies indicate that young adults who are undergoing a critical time of personal development (e.g., family formation, education, and career-building) reported higher levels of loneliness and anxiety at the beginning of the pandemic [5, 10, 14, 15]. Current evidence also suggests that individuals with a history or current diagnosis of chronic and/or mental illness – and who may thus require stricter social-isolation measures and/or be more sensitive to certain stressors, respectively – have higher levels of loneliness and anxiety compared to individuals without these conditions [10, 14, 16–18].

Many of the studies that report on adverse mental health effects during the COVID-19 pandemic have used cross-sectional study designs focusing on a specific short period during the pandemic, or used longitudinal designs with short follow-up times. Furthermore, most studies have focused on small study populations, which are non-representative samples of populations. We believe that continuously monitoring several mental health indicators in representative populations is essential for mental health surveillance, which can inform public health authorities and guide the governmental response to COVID-19 and future pandemics. Furthermore, such information may prove useful in the post-COVID-19 era; e.g., by showing which population subgroups might be most vulnerable to major events that impact entire countries.

In the current study, we investigated mental health landscapes over the course of the first 16 months of the COVID-19 pandemic in Danish adults. We define mental health as individuals’ general emotional, psychological, and social wellbeing, and we used six indicators covering various aspects of mental health (loneliness, anxiety, social isolation, quality of life, COVID-19-related worries, and the mental health scale) in this project. To track mental health landscapes over the course of the pandemic in Denmark, we used a unique population-based time-series of 43 timepoints, and we assessed differences in mental health time-series between population subgroups defined by gender, age, and self-reported pre-existing mental and/or chronic illness.

## Methods

### The Epinion time-series data

In collaboration with Epinion, a Danish consumer-research company, we utilized a repeated cross-sectional questionnaire data collected from the Danish population at multiple timepoints during the COVID-19 pandemic [17]. The main purpose of the collected data was to investigate the mental health impact of the pandemic on the Danish population. In this project, we used data collected between 20 March 2020 and 22 July 2021. Specifically, between 20 March and 16 April 2020, ~100 people from the adult (age 18 and above) general population answered an online survey every third day (i.e. an independent group responded to the survey at every sampling timepoint). Starting 26 April 2020, the sampling frequency changed to once a week until 25 June 2020, when data collection ceased for two months. The data collection was resumed on 10 September 2020, with ~250 people answering the survey every second week; by 22 July 2021, this resulted in a final study population of 8,261 participants from the adult general population. This study uses data from a total of 43 timepoints spanning 16 months of the COVID-19 pandemic. Due to survey design, the data had no missing values.

### Mental health indicators and population characteristics

To investigate the mental health consequences of the COVID-19 pandemic, investigators from the University of Copenhagen and their collaborators developed the Copenhagen Corona-Related Mental Health Questionnaire [17]. Basic sociodemographic information, such as age, self-identified gender, and prior diagnosis of chronic and/or mental illness (yes/no), was collected. Feelings of worries, and social isolation, were collected on 1-10 Likert scales, higher values representing worse levels. To measure quality of life, we used the adaptation of the Cantril Ladder scale [19], where individuals rated their quality of life on a 1-10 Likert scale, higher values representing better quality of life. Feelings of loneliness were assessed using the University of California, Los Angeles Three-Item Loneliness Scale (UCLA T-ILS) [20], and responses (1-3 Likert scale) for the three questions were tallied to generate a total score ranging from 3 to 9; here, higher scores represented higher levels of loneliness. Anxiety was assessed using the anxiety subscale of the Common Medical Disorder Questionnaire (CMDQ-ANX) [21]. Four questions were asked (1-5 Likert scale) and responses were tallied to generate a total score ranging from 4 to 20 in which higher scores represented higher levels of anxiety. We also utilized the mental health scale based on the core questions developed by investigators at Johns Hopkins University [22]. Here, responses (1-4 Likert scale) to five questions were tallied to generate a total score ranging from 5 to 20 in which higher scores represented poorer levels of mental health. All survey questions utilized for this project are described in detail in Text S1.

### Statistical analysis

**Fig. 1:**
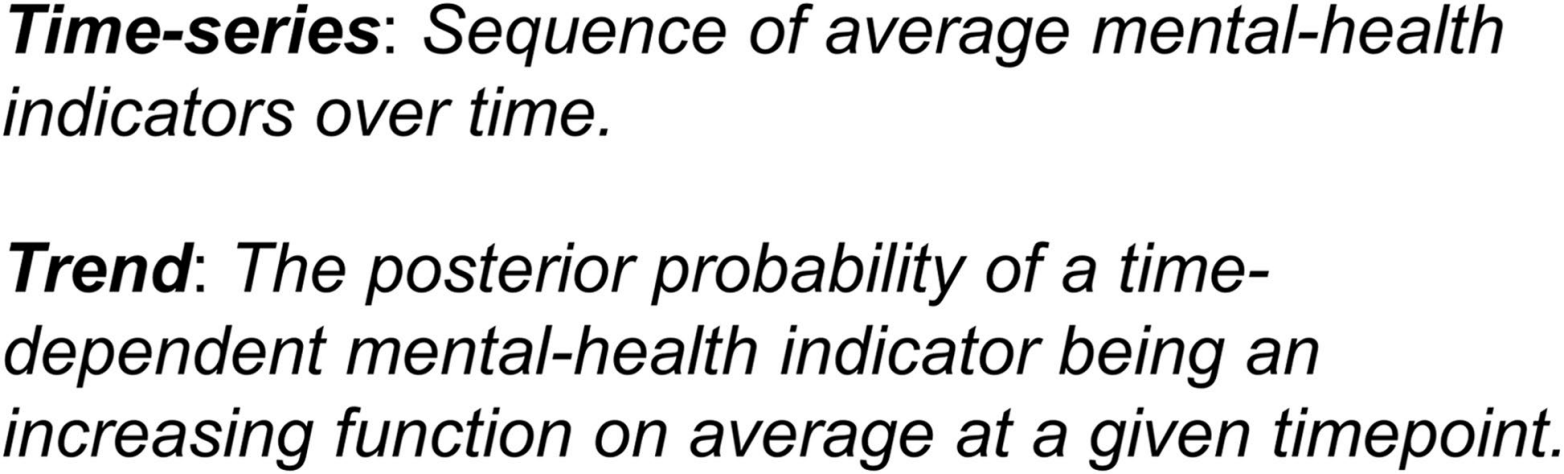
Definitions of *time-series* and *trends*

Weighting using the raking method, accounting for gender, age, and the regional composition of Denmark (extracted from Statistics Denmark, the Danish national statistical database) was performed [23]. In brief, the raking procedure upweighs individuals who are underrepresented compared to the national statistics, and downweighs those who are overrepresented. This procedure was undertaken at each timepoints separately, resulting in 43 comparable samples that are each weighted to represent the Danish population. We estimated *time-series* of means for all mental health indicators with Gaussian process regression [24] using the Exponential Squared covariance function as a prior. The likelihood was a t-distribution with the degree of freedom as a free parameter, and the scale parameter fixed at the standard error for each of the observed time averages. Visualizations were given as the observed means along with the model-based posterior time averages with accompanying 95% credible intervals and 95% percentiles of the posterior predictive distribution at the observation times. We used the Trend Direction Index (TDI) to assess *trends* in the mean mental health indicators throughout the study period. The TDI quantifies the posterior probability of a time-dependent outcome being an increasing function on average at a given timepoint, conditional on the observed data [25]. In other words, a trend is considered positive if the slope of the mean development over time (as defined by the first-order derivative) is positive, and a TDI above 50% indicates that the mean is more likely to be increasing than decreasing at that point in time. Hence, a TDI close to 100% indicates that the underlying time dynamics of a specific mental health indicator are increasing on average, a TDI close to 0% indicates that the underlying time dynamics are decreasing on average, and a TDI of 50% reflects complete uncertainty about the state of average monotonicity. Figure 1 describes the terms “time-series” and “trends” as the two main ways results are presented in this report. Of note, the authors refrain from using the term “trajectory” when describing average mental health indicators over time, as means over time were estimated using a repeated cross-sectional study design.

Subgroup comparisons were conducted by stratifying the total population by gender (women/men), age categories (18-34 years/35-64 years/65+ years), previously diagnosed chronic conditions (yes/no), and previously diagnosed mental illness (yes/no).

## Results

**Fig. 2:**
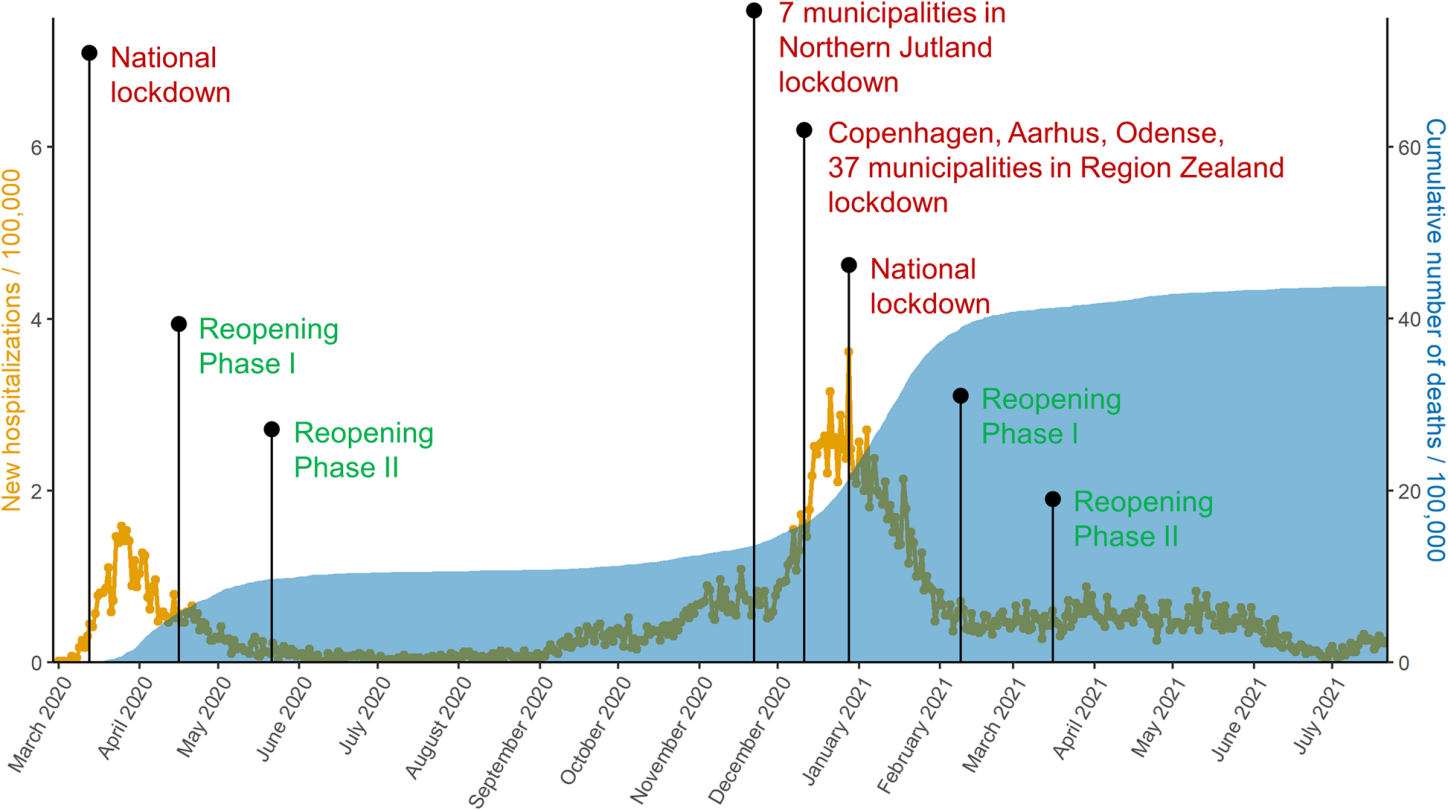
Governmental intervention timeline for Denmark. New hospitalisations / 100,000 population (orange) and cumulative number of deaths / 100,000 population (blue) are presented (28 February 2020 - 22 July 2021), and most important events related to lockdowns and reopenings are indicated on the figure

The first national lockdown was officially effective from 13 March 2020 (declared on 11 March 2020), while the second was introduced gradually but went into full effect on 25 December 2020. The most important easements of restrictions started on 15 April 2020 (Phase I) and 20 May 2020 (Phase II) for the first lockdown, and 6 February 2021 (Phase I) and 15 March 2021 (Phase II) for the second lockdown (Fig. 2). The number of new COVID-19-related hospitalizations and deaths peaked during March 2020, and between December 2020 – February 2021 (Fig. 2). Population characteristics are described in Table 1.

**Table 1:** Descriptive statistics (N=8,003)

	Total Population n (%)
**Gender**	
Women	4,348 (52.6)
Men	3,913 (47.4)
Total	8,261 (100)
**Age groups**	
18-34 years	2,329 (28.2)
35-64 years	3,468 (42.0)
65+ years	2,464 (29.8)
Total	8,261 (100)
**Chronic conditions**	
No	6,187 (74.9)
Yes	2,074 (25.1)
Total	8,261 (100)
**Mental illness**	
No	7,057 (85.4)
Yes	1,204 (14.6)
Total	8,261 (100)

### Mental health time-series and trends in the general population

**Fig. 3:**
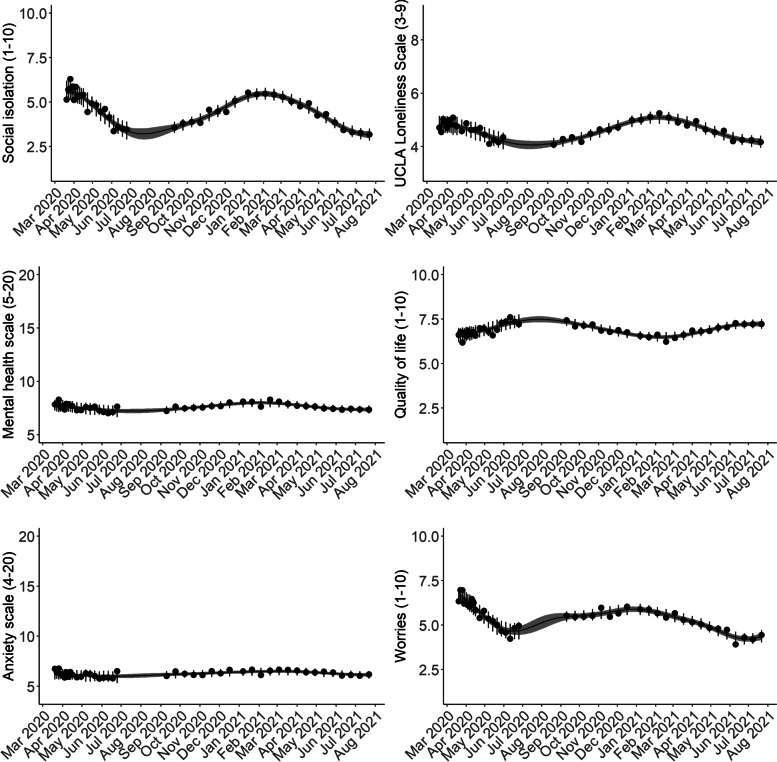
Posterior mean time-series of mental health indicators in Danish adults during the COVID-19 pandemic (N=8,261). The circles represent weighted means at each timepoint. The error bars at each circle show 95% intervals of the posterior predictive distribution. The continuous lines show the posterior mean over the entire study period, and the shaded regions are 95% credible intervals

**Fig. 4:**
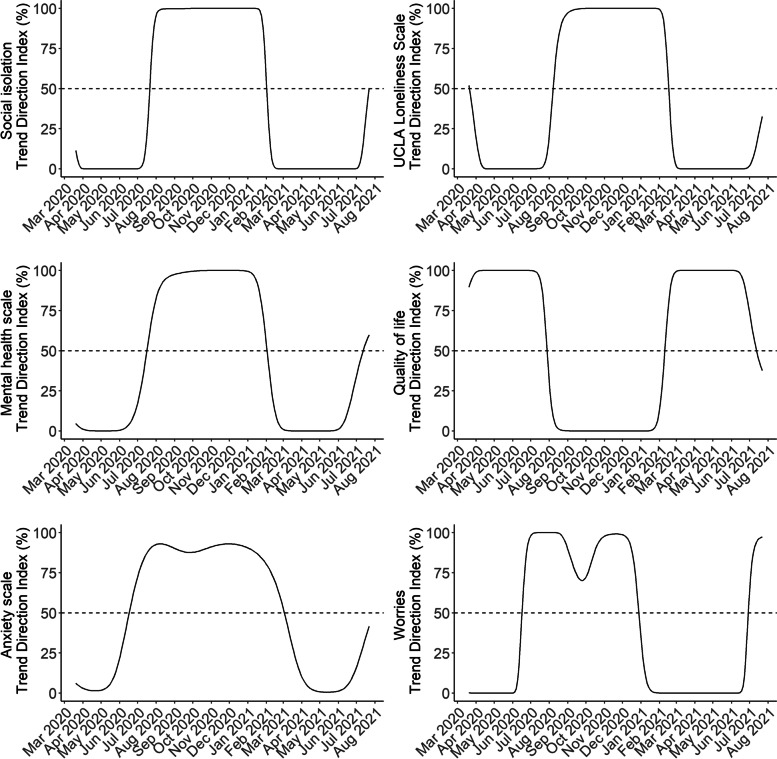
Trends direction indices in mental health time-series in Danish adults during the COVID-19 pandemic (N=8,261). The continuous lines show the trend direction indeces over the entire study period for the six mental health indicators. A TDI close to 100% indicates that the mental health indicator is increasing on average, a TDI close to 0% indicates that the mental health indicator is decreasing on average, and a TDI of 50% reflects complete uncertainty about the state of average monotonicity

Mean time-series for the six indicators are shown in Fig. 3. The time-series were similar for loneliness, social isolation, COVID-19-related worries, and quality of life. In general, we observed poorer mental health during the strict lockdown periods and better mental health during re-opening phases. For COVID-19-related worries, the worst levels over the entire course of the pandemic thus far were observed during the first lockdown (March – April 2020), while the worst levels of loneliness were observed during the second lockdown (December 2020 – February 2021). For social isolation and quality of life, levels were similar during the national lockdowns during March – April 2020, and December 2020 – February 2021. The anxiety and mental health scales (higher levels indicating poorer levels) were less reactive to lockdowns and re-openings; when interpreted on their full scales ranging between 4 and 20 and 5-20, respectively, both measures stayed relatively stable during the pandemic thus far. However, the trend plots (Fig. 4) indicate that, despite relatively stable levels, similar trends were observed for the anxiety and mental health scales compared to the four other indicators, with deteriorating trends before and during lockdowns and improving trends during re-opening phases. From observing the trend plots, we identified the time periods in which trends likely began to worsen/improve (i.e. when trend lines crossed the 50% TDI mark). Mental health indicators began to worsen between June – August 2020, with worries and anxiety shifting in early June 2020, while the other indicators beginning to worsen in July/August 2020. Levels of COVID-19-related worries were the first to shift to an improving time-series at the end of December 2020, shortly after the second national lockdown was implemented. The other mental health indicators began to improve between late January and early March 2021. It also appears that trends are shifting from certainly improving to deterioration again at the last couple of data collection timepoints where the pandemic is relatively under control in Denmark (July 2021).

### Mental health time-series in population subgroups

**Fig. 5:**
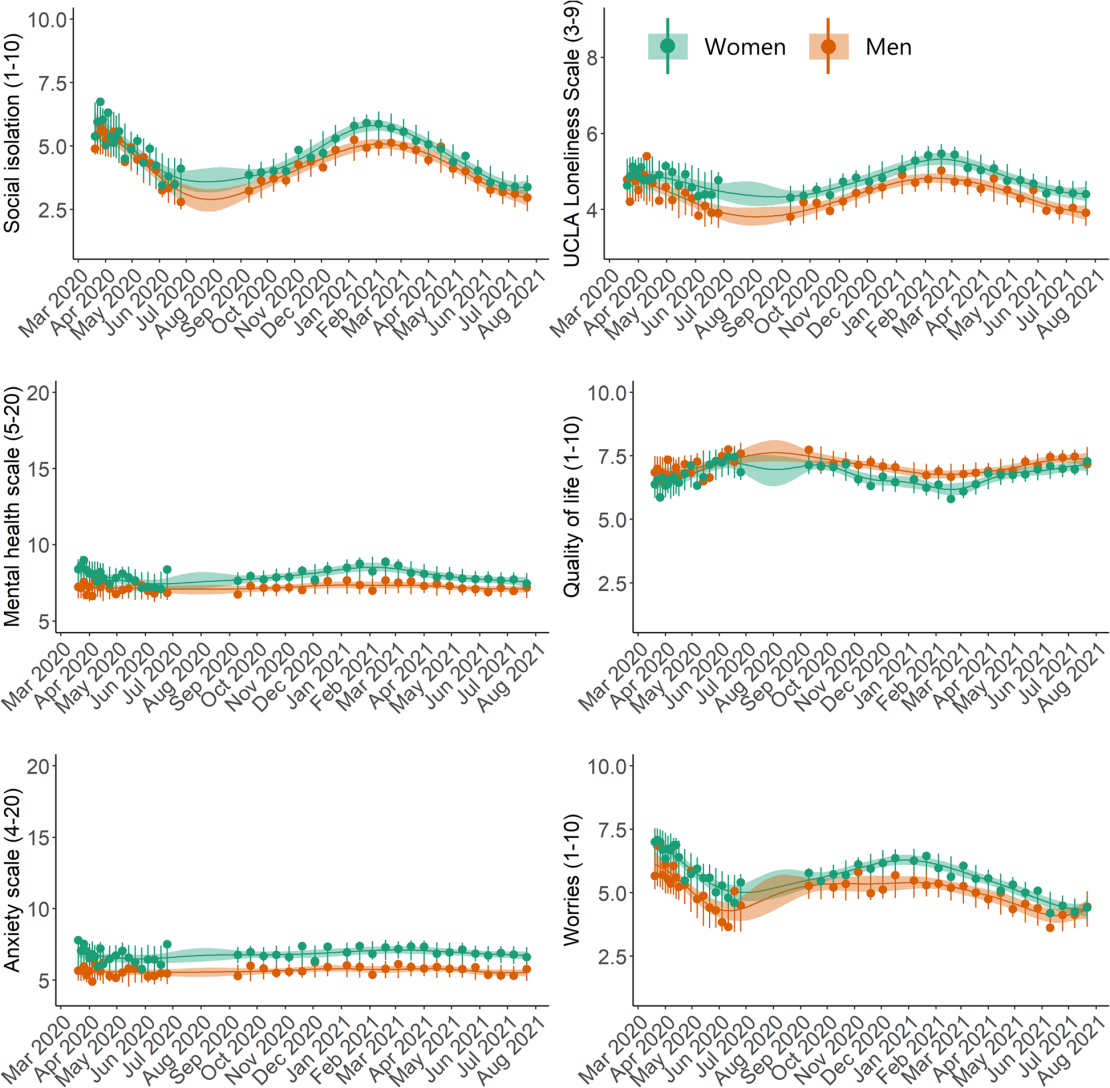
Posterior mean time-series of mental health indicators in Danish adults during the COVID-19 pandemic, stratified by gender (N=8,261). The circles represent weighted means at each timepoint. The error bars at each circle show 95% intervals of the posterior predictive distribution. The continuous lines show the posterior mean over the entire study period, and the shaded regions are 95% credible intervals

**Fig. 6:**
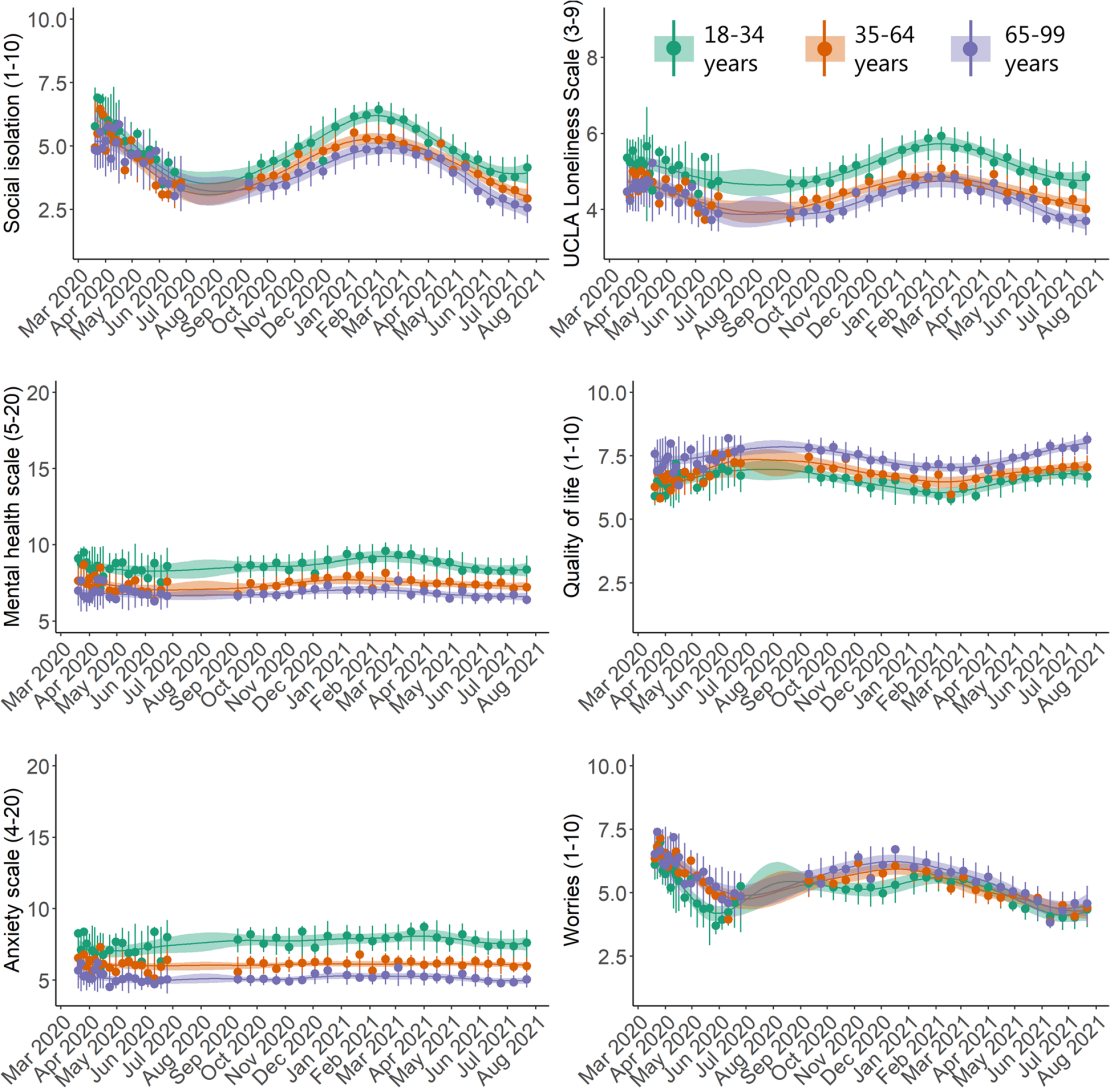
Posterior mean time-series of mental health indicators in Danish adults during the COVID-19 pandemic, stratified by age groups (N=8,261). The circles represent weighted means at each timepoint. The error bars at each circle show 95% intervals of the posterior predictive distribution. The continuous lines show the posterior mean over the entire study period, and the shaded regions are 95% credible intervals

As seen in the gender-stratified (Fig. 5) and age-stratified (Fig. 6) time-series, both women and those in the youngest age group (<34 years) demonstrated poorer mental health during the entire study period for all mental health indicators, with a few exceptions. An important exception is COVID-19-related worries where the youngest group showed more beneficial levels. Differences between age groups in the social isolation, loneliness, and the general mental health scale time-series became most pronounced during the second lockdown (early 2021). Similarly, time-series differences between men and women in the social isolation, loneliness, quality of life, and the mental health scales became larger during the second lockdown compared to the first lockdown.

**Fig. 7:**
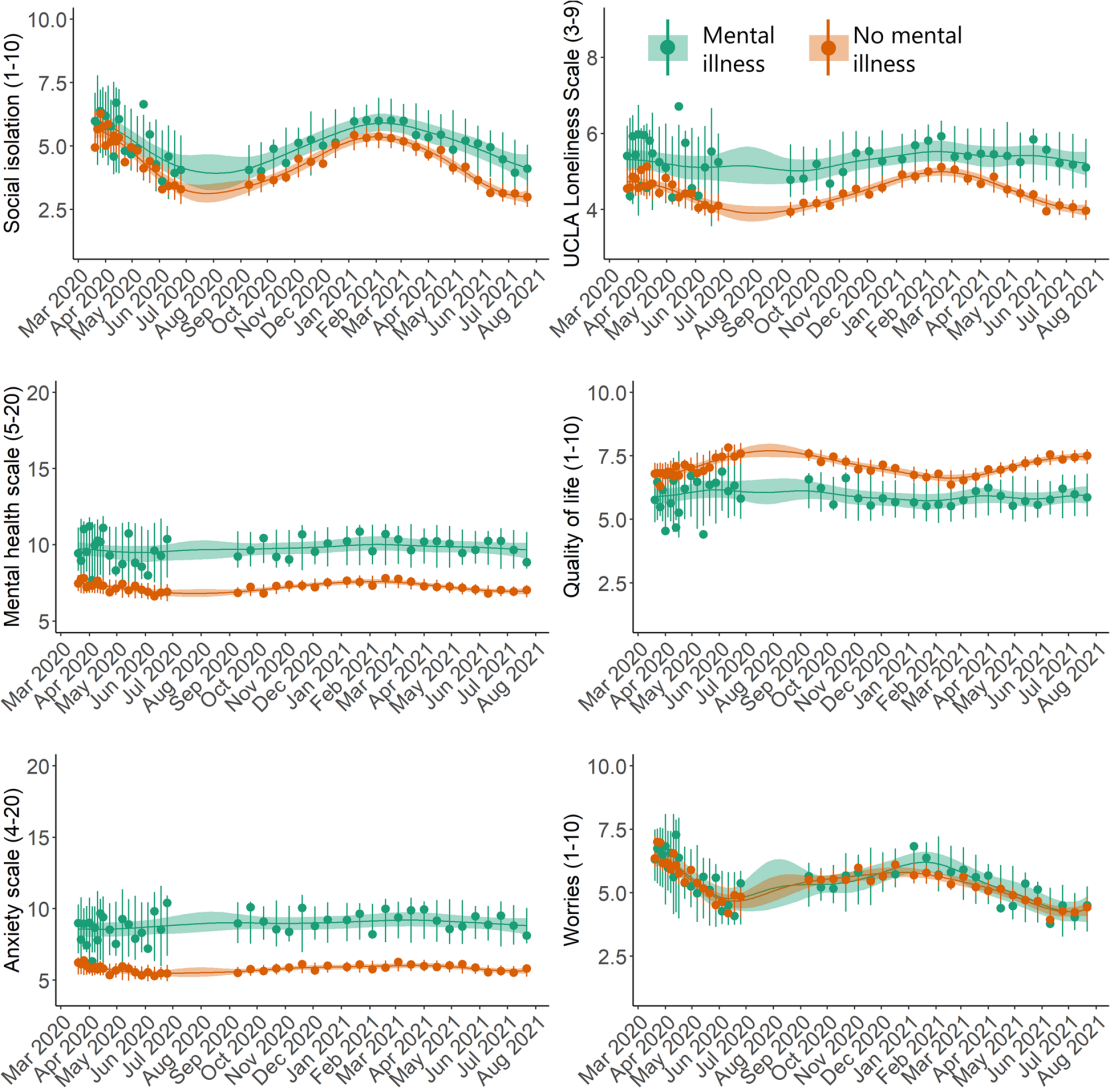
Posterior mean time-series of mental health indicators in Danish adults during the COVID-19 pandemic, stratified by previously diagnosed mental illness (N=8,261). The circles represent weighted means at each timepoint. The error bars at each circle show 95% intervals of the posterior predictive distribution. The continuous lines show the posterior mean over the entire study period, and the shaded regions are 95% credible intervals

**Fig. 8:**
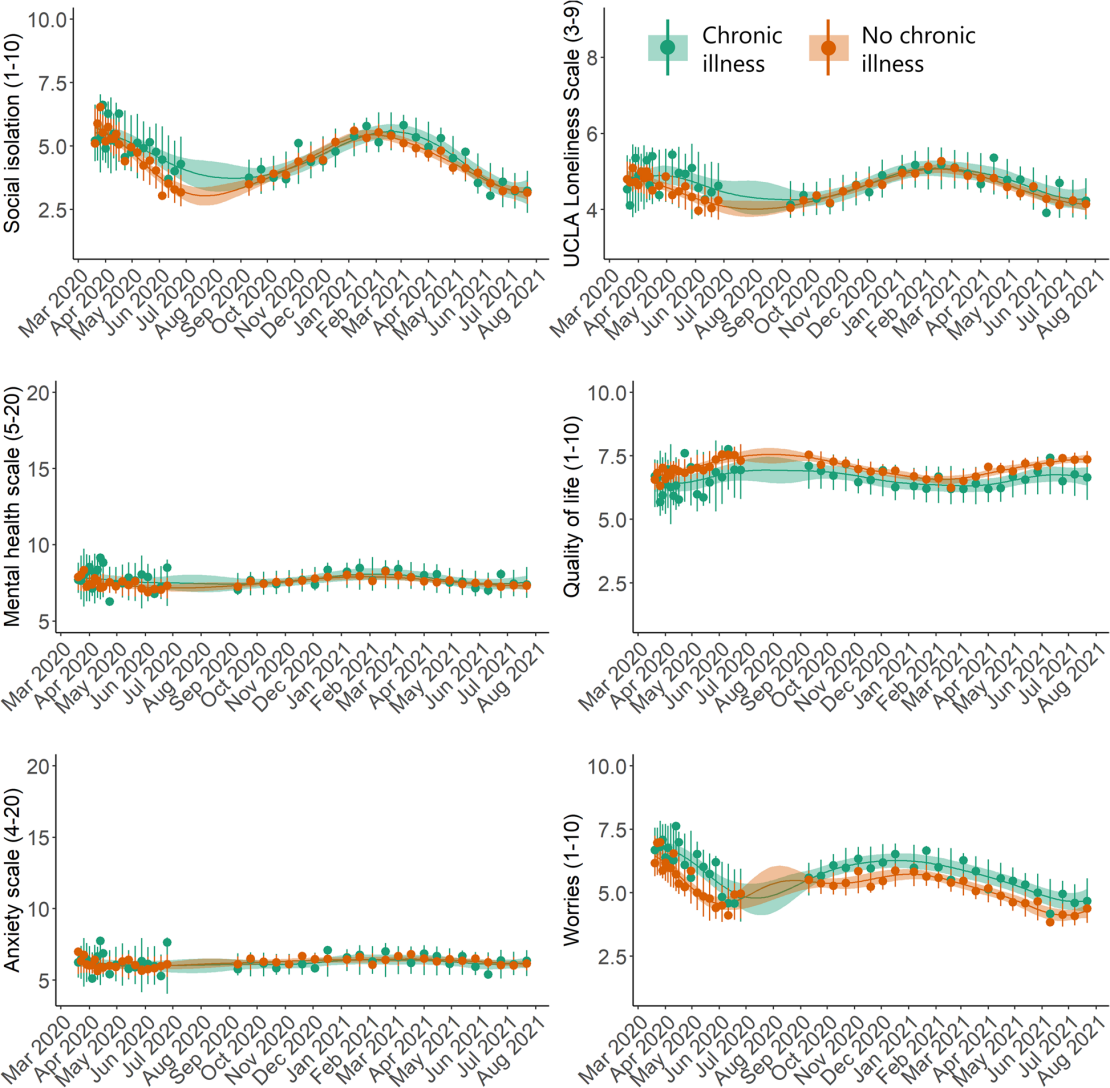
Posterior mean time-series of mental health indicators in Danish adults during the COVID-19 pandemic, stratified by previously diagnosed chronic illness (N=8,261). The circles represent weighted means at each timepoint. The error bars at each circle show 95% intervals of the posterior predictive distribution. The continuous lines show the posterior mean over the entire study period, and the shaded regions are 95% credible intervals

Individuals with self-reported previously diagnosed mental illness on average scored poorer on all mental health indicators except for worries during the entire period compared to those without mental illness (Fig. 7). With respect to loneliness, anxiety, quality of life, and the mental health scale, those with previously diagnosed mental illness demonstrated a generally constant, non-reactive time-series over the study period. With regards to individuals with self-reported previously diagnosed chronic illness, they reported poorer mean time-series of worries, quality of life, loneliness, and social isolation compared to those without chronic illness; however, for loneliness and social isolation, differences in time-series (non-overlapping 95% credible intervals) were only observed between April – July 2020 when the first lockdown and re-opening occurred (Fig. 8).

## Discussion

In this article, we investigated time-series of mental health indicators and their trends in Denmark over the first 16 months of the COVID-19 pandemic using a large, nationally representative population, and high-resolution surveillance data. Specifically, we jointly monitored time-trends of loneliness, anxiety, social isolation, quality of life, COVID-19-related worries, and a general mental health scale in a comprehensive fashion. We also investigated differences between population subgroups defined by gender, age, and self-reported previously diagnosed chronic and/or mental illness. The assessment of subgroup time-series have enabled us to identify subgroups that demonstrated more reactive patterns compared to others, highlighting those who might be especially vulnerable during the pandemic [26, 12, 27–29].

Not surprisingly, and as demonstrated previously [10], our findings indicate poorer mental health indicators during the strict lockdown periods, and better outcomes during reopening phases. Upon closer inspection, the improving trends in mental health indicators during May/June 2020 appeared to decline around July/August 2020 and continued to worsen until early 2021. Another shift in trends was observed around February 2021; at this time, most of the indicators began to improve. This suggests that shifts in trends towards improving or declining mental health loosely align with the initial signs in shifts towards lockdowns (introduction of mandatory facemasks in August 2020) and reopening phases (reopening of primary schools in February 2021). However, further qualitative or mixed-method studies are warranted to take a closer look into mechanisms through which the pandemic elicited a mental health response in the population.

Subgroup comparisons indicate that women, young adults, and people with previously diagnosed chronic and/or mental illness had poorer mental health during the pandemic thus far. As expected and previously observed and discussed elsewhere [12, 30], those with pre-existing mental illness in general had scored worse on all mental health indicators compared to those without mental illness. However, this group also appeared to be less reactive to pandemic severity and/or restrictions and re-openings compared to those without pre-existing mental illness. This is reflected in their relatively stable time-series with less pronounced peaks compared to the time-series of other groups, especially when considering loneliness, the mental health scale, and quality of life. This phenomenon could have resulted from built up resilience, or the delicate balance between the negative impacts of the pandemic and some of the positive aspects it generated; e.g. increased family or social support [31, 32], or a slower-paced world that provided novel coping strategies for those with pre-existing mental illness [33]. On the other hand, those with pre-existing mental illness would already have poorer levels of mental health indicators prior to the pandemic, and it is also possible that this group is under pharmacological or cognitive behavioural treatment that increases their capability to cope with stressors in general. Further research is warranted to gain a comprehensive understanding of the specific factors that increased the resilience of this population subgroup. Understanding these support mechanisms may help rethink public health interventions.

Those with pre-existing chronic illness did not follow the same time-series as those with mental illness. Although they reported poorer mental health levels during the first lockdown compared to individuals without chronic illness, these differences disappeared for the remainder of the pandemic thus far for loneliness and social isolation. We hypothesize that this may be due to initial uncertainties related to the virus and, as information rapidly accumulated during the first six months of the pandemic [34, 35], this population subgroup gained a better understanding of their individual risk and did not feel more isolated, or lonely compared to those without chronic illness. Gradual improvement of mental health indicators in response to easements in restrictions in those with chronic illness have been reported before [36]; while our study partly confirms these findings, in terms of level of worries and quality of life, those with chronic illness show a consistently poorer time-series for the entire course of the pandemic thus far compared to those without pre-existing chronic conditions.

Similar to those with pre-existing conditions, women and those in the youngest age group also scored worse with regards to mental health indicators prior to the pandemic, and these differences were sustained throughout the pandemic. While differences in levels of mental health indicators were more pronounced during the first lockdown between those with and without pre-existing chronic conditions, for strata defined by age groups and gender, the greatest differences were observed during the second lockdown. Reasons for this could be related to a COVID-19-related mental fatigue that seemed to impact those subgroups that had already demonstrated overall poorer levels throughout the pandemic.

To estimate time-series, we utilized repeated cross-sectional design with data collected at 43 timepoints. The greatest drawback of this study design is that it does not reflect differences over time in the same individuals but pools estimates for independent sets of individuals sampled at each timepoint. However, by utilizing a nationally representative sample at each timepoint, this approach avoids bias due to loss to follow-up, which is an extremely common methodological drawback in longitudinal cohort studies [26]. For this reason, the sampling and analytical approach in our study is appropriate for surveillance of time trends in mental health indicators during, e.g., prolonged lockdown periods. Although there are reports from large-scale longitudinal studies that have evaluated the mental health response of populations during the pandemic [12, 27–29], a concern is that individuals whose mental health is most negatively impacted by the pandemic and the associated lockdowns might be more likely to drop out during follow-up. Furthermore, this bias would become increasingly pronounced with longer follow-up times [26], making a strong case for our approach presented here. Also, we could not identify studies of any design that tracked mental health indicators for as long and as frequently as our study.

We declare a number of limitations. First, it is possible that people who are less negatively impacted by the pandemic may be more prone to answer online questionnaires. Furthermore, those in the oldest age group who have better cognitive functioning and technological competences might be more likely to respond to the survey; conversely, frail and institutionalized older people might be underrepresented in our data [17]. These potential selection biases would result in more optimistic estimates compared to the true levels in the population. Fundamentally, average levels of mental health indicators are likely to be worse than reported here. Second, we lack information about pre-pandemic levels of the six mental health indicators. This limitation makes it difficult to estimate the initial impact of the COVID-19 pandemic on the Danish population’s mental health in the January-March 2020 period; however, some of the highest levels of anxiety, loneliness, feelings of worries, and social isolation were observed during the first few days of data collection, which indicates a strong mental health response – kind of a shock effect of the pandemic. Third, different countries are characterized by a different COVID-19 incidence and death rates, and there are international variations in governmental restrictions. However, our previous research indicates that the initial mental health responses to the pandemic were consistent across several western and northern European countries, suggesting similar patterns despite variations in governmental strategies [10]. Regardless, we advise caution in generalizing our results, and we encourage further research on long-term time trends of mental health indicators during the pandemic worldwide. Fourth, all mental health indicators are self-reported, and thus prone to bias. However, the use of validated scales for loneliness, and anxiety reduces the chance of measurement bias; recall bias is also limited, as only two of the six mental health indicators require participants to use recall, and the recall period itself is short. Last, there is evidence of seasonal fluctuation in mental health indicators [37]. Using our data it was not possible to separate the impact of the pandemic, associated governmental interventions, and such seasonal factors; further studies are warranted to explore these relationships.

## Conclusions

Our most significant finding is that those with pre-existing mental illness have shown poor but stable levels of mental health during the course of the pandemic thus far. These individuals appear to be less impacted by the determinants that result in fluctuating time-series amongst the general population and most of the other groups that we examined. In line with previous recommendations, we call for continued support of individuals who appear to be more reactive to the pandemic and consistently demonstrate greater susceptibility to poor mental health outcomes as the pandemic continues, as well as increased efforts to contain the virus and address the negative impact of the pandemic and its associated lockdowns on mental health.

## Supplementary Information

**Additional file 1.**

**Additional file 2.**

## Data Availability

Due to ethical reasons, we are not able to share individual-level data. Regarding potential opportunities for collaboration, please contact the corresponding authors.
