# Supplementary material for: Time trends in mental health indicators during the initial 16 months of the COVID-19 pandemic in Denmark
Source: BMC Psychiatry. 2022 Jan 10;22:25. doi: 10.1186/s12888-021-03655-8 (PMC8743441; doi:10.1186/s12888-021-03655-8)
Supplement: Supplementary file 2 — Additional file 2. [file 12888_2021_3655_MOESM2_ESM.docx]

Supplemental Text 1. **CCHM Survey questions**

**Gender**

What is your gender?

Female/Male/Other/Prefer not to answer

**Age**

What is your age?

Numeric

**Chronic conditions**

Do you have any chronic health conditions?

Yes/No

**Mental illness**

Has a doctor or other healthcare provider ever told you that you have a mental health condition?

Yes/No

**COVID-19-related worries**

On a scale from 1 to 10, how worried are you about the corona crisis?

Likert scale 1-10 (1 = not at all, 10 = extremely)

**Social isolation**

On a scale from 1 to 10, how socially isolated do you feel right now?

Likert scale 1-10 (1 = not at all, 10 = extremely)

**Quality of life**

On a scale from 1 to 10, how would you rate your quality of life right now?

Likert scale 1-10 (1 = terrible, 10 = excellent)

**Loneliness (UCLA T-ILS)**

The following sentences describe how you may feel. State how often you feel as described.

1. How often do you feel that you miss having company?

2. How often do you feel left out?

3. How often do you feel isolated?

Likert scale 1-3 for all three questions (1 = Almost never or never, 2 = Sometimes, 3 = Often). Questions responses tallied to generate a total score ranging from 3-9.

**Mental health scale (Johns Hopkins University)**

In the past week, how often have you:

1. Felt nervous, anxious, or on edge?

2. Felt depressed?

3. Felt lonely?

4. Felt hopeful about the future?

5. Had physical reactions, such as sweating, trouble breathing, nausea, or a pounding heart, when thinking about your experience (e.g., social distancing, loss of income/work, concerns about infection) with the coronavirus?

Likert scale 1-4 for all five questions (1 = Rarely or none of the time (less than 1 day), 2 = Some or a little of the time (1-2 days), 3 = Occasionally or a moderate amount of time (3-4 days), 4 = Most or all of the time (5-7 days)). Fourth question reversed and questions responses tallied to generate a total score ranging from 5-20.

**Anxiety (CMDQ-ANX)**

During the last week, how much were you bothered by:

1. Feeling suddenly scared for no reason?

2. Nervousness or internal instability?

3. Moments of terror or panic?

4. Feeling like you worry too much?

Likert scale 1-5 for all four questions (1 = Not at all, 2 = A little, 3 = Moderately, 4 = Quite a bit, 5 = Most of the time). Fourth question reversed and questions responses tallied to generate a total score ranging from 4-20.
